# Head-to-head comparison of aggressive conventional therapy and three biological treatments and comparison of two de-escalation strategies in patients who respond to treatment: study protocol for a multicenter, randomized, open-label, blinded-assessor, phase 4 study

**DOI:** 10.1186/s13063-017-1891-x

**Published:** 2017-04-04

**Authors:** Daniel Glinatsi, Marte S. Heiberg, Anna Rudin, Dan Nordström, Espen A. Haavardsholm, Bjorn Gudbjornsson, Mikkel Østergaard, Till Uhlig, Gerdur Grondal, Kim Hørslev-Petersen, Ronald van Vollenhoven, Merete L. Hetland

**Affiliations:** 1 Copenhagen Center for Arthritis Research (COPECARE), Center for Rheumatology and Spine Diseases, Centre of Head and Orthopaedics, Rigshospitalet, Nordre Ringvej 57, DK-2600 Glostrup, Denmark; 2 Department of Rheumatology, Diakonhjemmet Hospital, Box 23 Vinderen, 0219 Oslo, Norway; 3 Clinical Rheumatology Research Centre, Sahlgrenska University Hospital, Gröna Stråket 14, 413 45 Gothenburg, Sweden; 4 Department of Rheumatology and Inflammation Research, The Sahlgrenska Academy at Gothenburg University, Box 480, 405 30 Gothenburg, Sweden; 5 Helsinki University Central Hospital and University of Helsinki, Division of Internal Medicine, Stenbäcksgatan 9 A, FIN-00290 Helsinki, Finland; 6 Centre for Rheumatology Research, University Hospital, v/Hringbraut, 101, Reykjavik, Iceland; 7 Faculty of Medicine, University of Iceland, Reykjavik, Iceland; 8 Department of Clinical Medicine, Faculty of Health and Medical Sciences, University of Copenhagen, Copenhagen, Denmark; 9 Department of Rheumatology, University Hospital, Fossvogur, 101, Reykjavik, Iceland; 10 National Advisory Unit for Rehabilitation in Rheumatology, Department of Rheumatology, Diakonhjemmet Hospital, Box 23 Vinderen, 0319 Oslo, Norway; 11 Department of Rheumatology, King Christian 10th Hospital for Rheumatic Diseases, Toldbodgade 3, 6300 Graasten, Denmark; 12 Institute of Regional Health Research, University of Southern Denmark, Graasten, Denmark; 13 Unit for Clinical Therapy Research, Inflammatory Diseases, The Karolinska Institutet, 171 76 Stockholm, Sweden

**Keywords:** Rheumatoid arthritis, Early treatment, Biological treatment, Aggressive conventional treatment, Sustained remission, De-escalation strategy

## Abstract

**Background:**

New targeted therapies and improved treatment strategies have dramatically improved the outcomes of patients with rheumatoid arthritis (RA). However, it is unknown whether different early aggressive interventions can induce stable remission or a low-active disease state that can be maintained with conventional synthetic disease-modifying antirheumatic drug (csDMARD) therapy, and whether they differ in efficacy and safety. The Nordic Rheumatic Diseases Strategy Trials And Registries (NORD-STAR) study will assess and compare (1) the proportion of patients who achieve remission in a head-to-head comparison between csDMARD plus glucocorticoid therapy and three different biological DMARD (bDMARD) therapies with different modes of action and (2) two de-escalation strategies in patients who respond to first-line therapy.

**Methods/design:**

In a pragmatic, 80–160-week, multicenter, randomized, open-label, assessor-blinded, phase 4 study, 800 patients with early RA (symptom duration less than 24 months) are randomized 1:1:1:1 to one of four different treatment arms: (1) aggressive csDMARD therapy with methotrexate + sulphasalazine + hydroxychloroquine + i.a. glucocorticoids (arm 1A) or methotrexate + prednisolone p.o. (arm 1B), (2) methotrexate + certolizumab-pegol, (3) methotrexate + abatacept, or (4) methotrexate + tocilizumab. The primary clinical endpoint is the proportion of patients reaching Clinical Disease Activity Index (CDAI) remission at week 24. Patients in stable remission over 24 consecutive weeks enter part 2 of the study earliest after 48 weeks. Patients not achieving sustained CDAI remission over 24 consecutive weeks, exit the study after 80 weeks. In part 2, patients are re-randomized to two different de-escalation strategies, either immediate or delayed (after 24 weeks) tapering, followed by cessation of study medication. All patients remain on stable doses of methotrexate. The primary clinical endpoint in part 2 is the proportion of patients in remission (CDAI ≤2.8) 24 weeks after initiating treatment de-escalation. Radiographic assessment will be performed regularly throughout the trial, and blood and urine samples will be stored in a biobank for later biomarker analyses.

**Discussion:**

NORD-STAR is the first investigator-initiated, randomized, early RA trial to compare (1) csDMARD and three different bDMARD therapies head to head and (2) two different de-escalation strategies. The trial has the potential to identify which treatment strategy to apply in early RA to achieve the best possible outcomes for both patients and society.

**Trial registration:**

NCT01491815 and NCT02466581. Registered on 8 December 2011 and May 2015, respectively.

EudraCT: 2011-004720-35

**Electronic supplementary material:**

The online version of this article (doi:10.1186/s13063-017-1891-x) contains supplementary material, which is available to authorized users.

## Background

Rheumatoid arthritis (RA) is a common inflammatory disorder with a reported prevalence of 0.5–1% of the population [1]. It is characterized by progressive inflammatory arthritis with joint swelling and tenderness. Over time, structural joint damage evidenced by radiographic progression may occur and joint function diminishes [2, 3]. The economic burden of RA is substantial with high annual direct and indirect costs, mainly associated with lost income from work [4].

During the few last decades, new pharmacologic treatments and treatment strategies have been introduced, which have dramatically improved the outcomes in RA. Therapy with conventional synthetic disease-modifying antirheumatic drugs (csDMARD), most commonly methotrexate in combination with glucocorticoids, are still first-line treatment. Combining methotrexate with other csDMARDs may further increase their clinical effect [5]. However, there are limited convincing data that combination treatment is superior when it comes to the prevention of radiographically demonstrable damage.

In the Fin-RACo trial, sulphasalazine monotherapy, stepwise changed to other csDMARD monotherapy if there is an inadequate response and supplemented with orally administered prednisolone if needed, was compared with combination therapy with methotrexate, hydroxychloroquine, sulphasalazine and orally administered prednisolone. A significantly higher number of patients were in remission and showed less erosive disease in the combination therapy group after 1 and 2 years [6]. Follow-up results at 5 and 11 years showed that clinical and radiographic outcomes of the patients in the combination therapy group continued to be superior to the monotherapy group [7, 8]. The CIMESTRA trial achieved 34–43% Disease Activity Score (DAS) remission after 1 year, increasing to 50–51% and 76–80% after 2 and 5 years, respectively, in early RA by aggressive use of glucocorticoid injections administered via the intra-articular (i.a.) route in addition to either monotherapy with methotrexate or combination therapy with methotrexate and cyclosporine A [9–11]. The high remission rates with this strategy were confirmed in the 2-year OPERA trial, and in both studies radiographic progression was minimal [12].

Tight disease control with frequent patient visits and the application of a treat-to-target approach is another way to improve outcomes. This was demonstrated in the TICORA study in which patients with RA of less than 5 years’ duration were treated with csDMARD therapy and were randomized to either intensive management or routine care. In the tight control group, more patients reached remission and the radiographically demonstrable erosive progression rate tended to be reduced in the tight control group compared to the routine care group [13].

The effect of biological disease-modifying antirheumatic drugs (bDMARDs) in combination with methotrexate in early RA is thoroughly documented in a number of large randomized controlled trials. Consistently, the tumor necrosis factor (TNF) inhibitors as well as other bDMARD treatments have shown superior clinical and radiographic results when given in combination with methotrexate compared to methotrexate alone [14–16]. However, bDMARD therapy is very expensive and associated with an increased rate of serious infections. Thus, according to the standing European League Against Rheumatism (EULAR) recommendations for the management of rheumatoid arthritis, bDMARD treatment should be added only after an initial trial of csDMARD treatment [17]. No previous studies of early RA have compared the efficacy and safety between csDMARD therapy and several bDMARD therapies with different modes of action in a head-to-head design.

The principle of early treatment is based on the concept of a therapeutic window of opportunity early in the disease course, and that a delay in starting DMARD therapy has a significant negative impact on the patient’s disease course and mortality [18]. Patients treated early have a significant reduction of radiographic progression, and patients with more aggressive disease seem to benefit most from early DMARD initiation [19]. Studies have shown that a substantial number of patients with established RA, who have previously failed csDMARD therapy, experience disease flare upon withdrawal of TNF inhibitors [20–23]. In patients with early RA, on the other hand, sustained biological-free remission may be achievable [24–26]. It is not known whether a more aggressive strategy, with the use of bDMARDs in the initial phase, can modulate the disease course more effectively, and further whether any particular drug mechanism of action works better in inducing remission that subsequently can be sustained with simpler means (induction-maintenance therapy).

The main objectives of the Nordic Rheumatic Diseases Strategy Trials And Registries (NORD-STAR) study are: (1) to assess and compare head to head the proportion of patients with early RA who achieve remission with aggressive csDMARD therapy and three bDMARD therapies with different modes of action and (2) to assess and compare two alternative de-escalation strategies in patients who respond to first-line therapy.

## Methods/design

### Study design

**Fig. 1:**
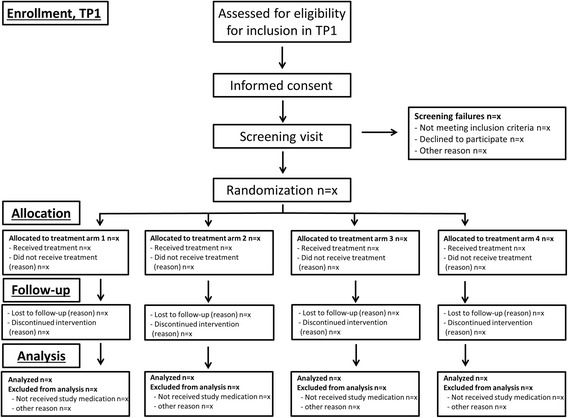
Flow chart of the phases in treatment part 1 (TP1) of the NORD-STAR study

**Fig. 2:**
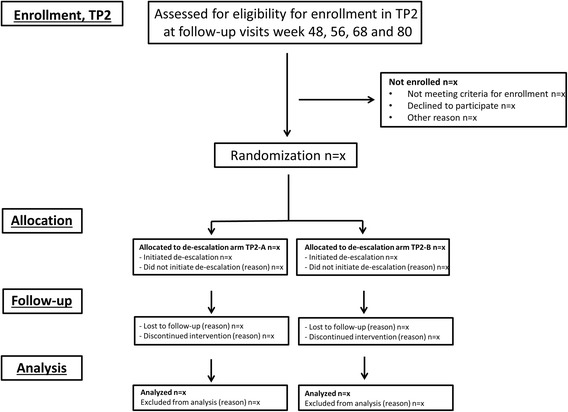
Flow chart of the phases in treatment part 2 (TP2) of the NORD-STAR study

The NORD-STAR study is a pragmatic, 80–160-week, multicentre, randomized, open-label, assessor-blinded, multiarm, parallel, phase 4 study in early RA, conducted at multiple centers in Denmark, Finland, Iceland, Norway and Sweden. Flow charts of the phases of the study are presented in Figs. 1 and 2, and a completed Standard Protocol Items: Recommendations for Interventional Trials (SPIRIT) Checklist for clinical trial protocols is presented in Additional file 1.

**Fig. 3:**
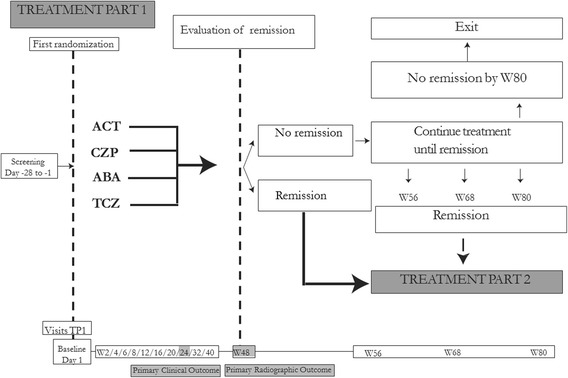
Overview of the NORD-STAR study, treatment part 1. Abbreviations: *TP1* treatment part 1, *ACT* aggressive conventional synthetic disease-modifying antirheumatic therapy, *TNF* tumor necrosis factor, *ABA* abatacept, *TCZ* tocilizumab, *W* week

**Fig. 4:**
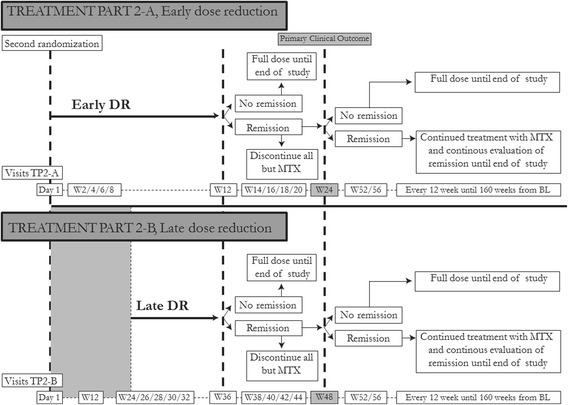
Overview of the NORD-STAR study, treatment parts 2 A and B. Abbreviations: *TP2* treatment part 2, *DR* dose reduction, *W* week, *MTX* methotrexate, *BL* baseline

The study consists of two treatment parts. In treatment part 1 (TP1, Fig. 3), aggressive csDMARD therapy is compared head to head with three different bDMARD therapies. In treatment part 2 (TP2, Fig. 4), two different de-escalation strategies are compared in patients who respond to the treatment.

### Randomization procedures

Randomization lists have been generated by the central study site several months before including the first patient in the NORD-STAR study, using an online computer program (https://www.randomizer.org/). At screening, the patient will receive a site-specific, unique patient number, which stays with the patient throughout the study. When a patient is found eligible, the central study site will be contacted by the local investigator via telephone or e-mail. Allocation of patients to study arm is done by the central study site at the time of inclusion (baseline of TP1) and to de-escalation strategy upon initiation of TP2. In TP1, patients are scheduled for a baseline visit where they are randomly allocated on a 1:1:1:1 ratio to one of four different treatment arms (Table 1). Stratification will be performed by country, gender and anti-citrullinated peptide antibody (ACPA) status (positive/negative). After allocation, a confirming receipt is sent to the local investigator via e-mail.

**Table 1:** Summary of the different treatment arms applied in the NORD-STAR study

All patients: MTX, starting at 10 or 15 mg/week, escalated by 5 mg/week to 25 mg/week within 4 weeks.	
To this is added 1 of 4 treatment arms below.	
*ARM 1A*: Aggressive csDMARD therapy followed by the investigators in Denmark and Finland: MTX + SSZ + HCQ + i.a. glucocorticoids	*SSZ*: 1 g/day (first week), 2 g/day (second week and on) *HCQ*: 35 mg/kg/week^a^ *i.a. injections of triamcinolonhexacetonid*: 20 mg/mL or equivalent (maximum 4 joints or 4 ml per visit). Injections must be given whenever clinically swollen joints are present. Priority to larger joints. Orally administered glucocorticoid is not allowed
*ARM 1B*: Aggressive csDMARD therapy followed by the investigators in Iceland, Norway and Sweden: MTX + p.o. glucocorticoids	*Prednisolone*: initially 20 mg/day, subsequently tapered in 9 weeks to 5 mg/day and discontinuation after 9 months. Glucocorticoid injections (i.a.) of triamcinolonhexacetonid, 20 mg/mL or equivalent is allowed when clinically indicated
*ARM 2*: bDMARD agent 1: MTX + certolizumab-pegol	*Certolizumab-pegol*: 400 mg s.c. given at 0, 2 and 4 weeks, thereafter 200 mg s.c. every other week. Glucocorticoid injections (i.a.) of triamcinolonhexacetonid, 20 mg/mL or equivalent is allowed when needed during the first 12 weeks. Thereafter, one to two injections (2 ml total or 40 mg) every 12 weeks. Orally administered glucocorticoid is not allowed
*ARM 3*: bDMARD agent 2: MTX + abatacept	*Abatacept*: 125 mg s.c. every week. Glucocorticoid injections (i.a.) of triamcinolonhexacetonid, 20 mg/mL or equivalent is allowed when needed during the first 12 weeks. Thereafter, one to two injections (2 ml total or 40 mg) every 12 weeks. Orally administered glucocorticoid is not allowed
*ARM 4*: bDMARD agent 3: MTX + tocilizumab	*Tocilizumab*: 8 mg/kg i.v. every 4 weeks or 162 mg s.c. every week. Glucocorticoid injections (i.a.) of triamcinolonhexacetonid, 20 mg/mL or equivalent is allowed when needed during the first 12 weeks. Thereafter, one to two injections (2 ml total or 40 mg) every 12 weeks. Orally administered glucocorticoid is not allowed

^a^In Denmark and Finland the patients receive the standard-of-care dose of 200 mg/day and 200–300 mg/day respectively, since other doses are not available

*Abbreviations*: *bDMARD* biological disease-modifying antirheumatic drugs, *csDMARD* conventional synthetic disease-modifying antirheumatic drugs, *MTX,* methotrexate, *SSZ* sulphasalazine, *HCQ* hydroxychloroquine, *i.a.* intra-articular, *s.c.* subcutaneous, *i.v.* intravenous, *p.o.* per os

### Assessment of disease activity and treatment response

Disease activity and treatment response are assessed by the Clinical Disease Activity Index (CDAI), which is a composite score of a 28 swollen joint count (SJC), a 28 tender joint count (TJC), patient global disease activity (assessed by the patient on a scale from 0 to 10 where 10 is maximal activity) and evaluator’s global disease activity (assessed by the evaluator on a scale from 0 to 10 where 10 is maximal activity). The CDAI score is interpreted as follows: remission: ≤2.8, low disease activity: >2.8 and ≤10, moderate disease activity: >10 and ≤22, high disease activity: >22.

### Treatment strategy in TP1 and TP2

In all treatment arms of TP1, the patients receive methotrexate, escalated to 25 mg/week within 1 month. To this is added either sulphasalazine + hydroxychloroquine + i.a. glucocorticoids (arm 1A, applied in Denmark and Finland) or orally administered prednisolone (arm 1B, applied in Iceland, Norway and Sweden) or one of three different bDMARD therapies (arm 2: certolizumab-pegol; arm 3: abatacept; arm 4: tocilizumab). Glucocorticoids i.a. are administered when clinically indicated (or for arm 1A, whenever a swollen joint is detected at a visit), but not 4 weeks prior to every time point for clinical outcome evaluation (weeks 20–24, 44–48, 52–56, 64–68 and 76–80 from baseline in TP1).

At 48 weeks from baseline, patients who have been in CDAI remission over the preceding 24 consecutive weeks will enter TP2. Patients not fulfilling this criterion will be re-assessed at weeks 56, 68 and 80. If the patient has not fulfilled CDAI remission over 24 consecutive weeks by week 80, the patient exits the study and the patient is scheduled for a follow-up telephone contact 12 weeks after termination.

Patients eligible for TP2 are randomized to either early (TP2-A) or late (TP2-B) dose reduction followed by discontinuation. In both arms, methotrexate is maintained at an unchanged dose. In TP2-A, de-escalation starts immediately upon entering TP2 and all bDMARDs and csDMARDs except methotrexate are tapered through 12 weeks and thereafter discontinued, provided that a flare has not occurred. In TP2-B, the same de-escalation starts after 24 additional weeks of full medication. Tapering will proceed as follows: dose of sulphasalazine, hydroxychloroquine and orally administered prednisolone will be halved and dose intervals of certolizumab-pegol, abatacept and tocilizumab will be doubled. If a flare occurs at any time point (i.e., patient has a CDAI >2.8), it is recommended to resume the full dose of study medication until the end of the study. Whether the patient should resume full medication or not will be decided by the local investigator at each site. Patients are followed until 80 weeks from inclusion in TP2. Glucocorticoids i.a. may be administered throughout TP2, but not 4 weeks prior to every time point for clinical outcome evaluation (TP2-A: weeks 8–12, 20–24 and 76–80 from the first day in TP2-A; TP2-B: weeks 20–24, 32–36, 44–48 and 76–80 from the first day in TP2-B).

If the patient fails to attend a scheduled visit, the study site will contact the patient via telephone or e-mail to schedule a new appointment.

Patients may withdraw from the study at any time and will be discontinued from the study if they do not receive a dose of study drug for three consecutive months. Patients will be withdrawn if any of the following occur: clinically significant abnormal laboratory results or adverse events (AEs), which rule out continuation of the study medication, as determined by the investigator; lack of efficacy (e.g., persisting DAS28 values >5.1, increasing DAS28 values or increasing core set variables), death, the patient becomes pregnant while on study medication, other illness that is not compatible with treatment according to the local investigator, failure to adhere to the protocol or a clinical course not acceptable within the normally applied paradigms of early RA between baseline and 6 months’ follow-up, as determined by the investigator.

**Fig. 5:**
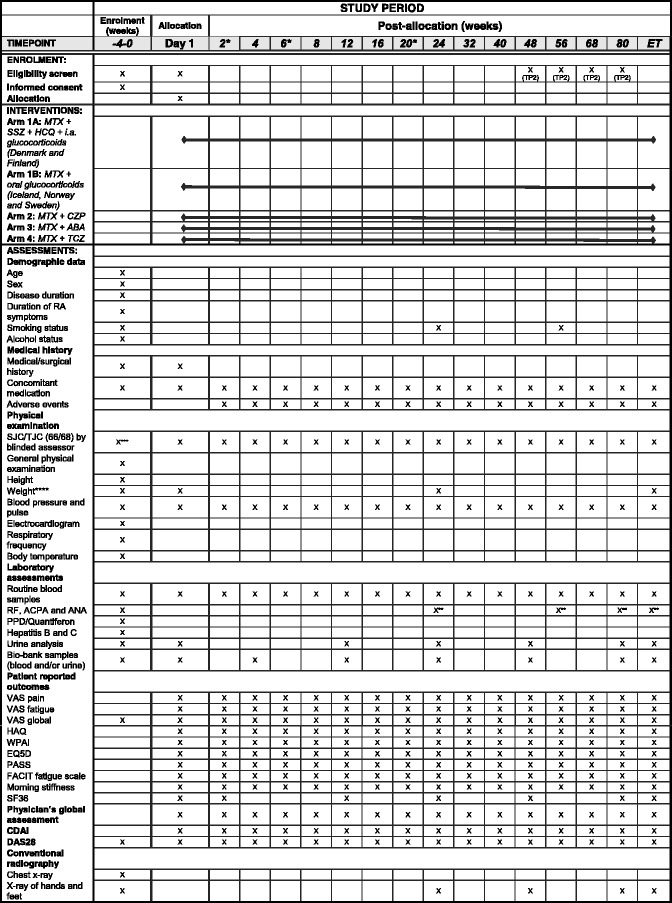
Schedule of enrollment, interventions and assessments in the NORD-STAR study, treatment part 1. *This visit may be replaced by a telephone contact. **Measured if clinically indicated. ***Blinding of joint-assessor is not necessary since the patient has not been randomized to a treatment arm at screening. ****If tocilizumab is given intravenously, the weight is measured at all visits. Abbreviations: *ET* early termination, *MTX* methotrexate, *SSZ* sulphasalazine, *HCQ* hydroxychloroquine, *i.a.* intra-articular, *CZP* certolizumab-pegol, *ABA* abatacept, *TCZ* tocilizumab, *RA* rheumatoid arthritis, *SJC* swollen joint count, *TJC* tender joint count, *RF* rheumatoid factor, *ACPA* anti-citrullinated protein antibodies, *ANA* anti-nuclear antibody, *PPD* purified protein derivate, *VAS* Visual Analogue Scale, *HAQ* Health Assessment Questionnaire, WPAI, Work Productivity Activity Impairment, *EQ5D* EuroQol 5 dimensions, *PASS*, Patient Acceptable Symptom State, FACIT Functional Assessment of Chronic Illness Therapy, SF36 Short Form 36, CDAI Clinical Disease Activity Index, DAS Disease Activity Score

**Fig. 6:**
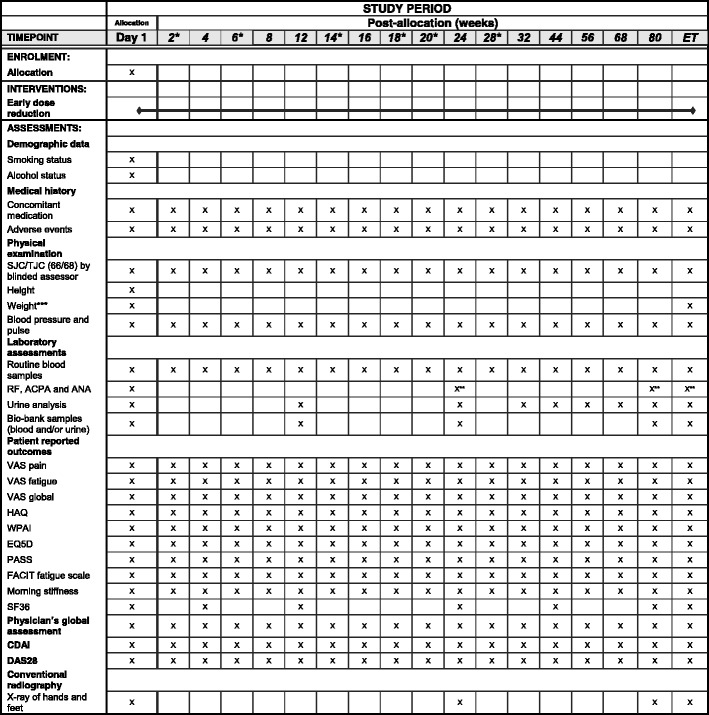
Schedule of enrollment, interventions and assessments in the NORD-STAR study, treatment part 2A. *This visit may be replaced by a telephone contact. **Measured if clinically indicated. ***If tocilizumab is given intravenously, the weight is measured at all visits. Abbreviations: *ET* early termination, *SJC* swollen joint count, *TJC* tender joint count, *RF* rheumatoid factor, *ACPA* anti-citrullinated protein antibodies, *ANA* anti-nuclear antibody, *VAS* Visual Analogue Scale, *HAQ* Health Assessment Questionnaire, *WPAI* Work Productivity Activity Impairment, *EQ5D* EuroQol 5 dimensions, *PASS* Patient Acceptable Symptom State, FACIT Functional Assessment of Chronic Illness Therapy, SF36 Short Form 36, CDAI Clinical Disease Activity Index, DAS Disease Activity Score

**Fig. 7:**
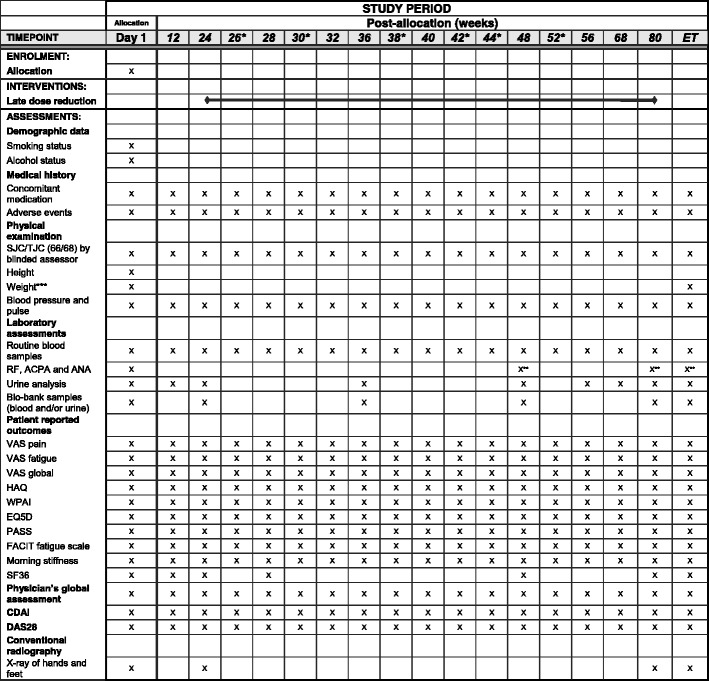
Schedule of enrollment, interventions and assessments in the NORD-STAR study, treatment part 2B. *This visit may be replaced by a telephone contact. **Measured if clinically indicated. ***If tocilizumab is given intravenously, the weight is measured at all visits. Abbreviations: *ET* early termination, *SJC* swollen joint count, *TJC* tender joint count, *RF* rheumatoid factor, *ACPA* anti-citrullinated protein antibodies, *ANA* anti-nuclear antibody, *VAS* Visual Analogue Scale, *HAQ* Health Assessment Questionnaire, *WPAI* Work Productivity Activity Impairment, *EQ5D* EuroQol 5 dimensions, *PASS* Patient Acceptable Symptom State, *FACIT* Functional Assessment of Chronic Illness Therapy, *SF36* Short Form 36, *CDAI* Clinical Disease Activity Index, *DAS* Disease Activity Score

If patients in treatment arm 1B have an unsatisfactory clinical course at the 6-month visit (or thereafter), the investigator will have the option of adding sulphasalazine and/or hydroxychloroquine to the treatment regimen, without precluding them to continue within the protocol. This is not possible for patients in other treatment arms. Since treatment recommendations for clinical practice advocates a treat-to-target approach in which treatment is evaluated after 3 months, it will not be considered a protocol violation if patients in the trial have their csDMARD therapy adjusted after, e.g., 3 months. If the patient at any stage of the trial terminates the study prematurely, the patient will be asked to complete an early termination visit (Figs. 5, 6 and 7). In addition, a follow-up telephone contact will be scheduled 12 weeks after the exit.

If a patient does not tolerate orally administered methotrexate at a dose of 25 mg/week, the investigator is allowed to reduce the dose or change to subcutaneously administered (s.c.) methotrexate. If methotrexate still cannot be tolerated, the investigator may replace methotrexate with either leflunomide or azathioprine. If this adjustment is not tolerated, patients in arms 2, 3 and 4 are allowed to remain on bDMARD treatment as monotherapy. Patients with persistent failure to tolerate the assigned drug(s) despite these adjustments will exit the study and receive standard-of-care treatment.

Acetaminophen, nonsteroidal anti-inflammatory drugs and tramadol analgesics are allowed as needed throughout the study.

### Patients

Patients diagnosed with early RA (symptom duration less than 24 months) will be recruited during routine visits at the rheumatology departments while they are DMARD-naïve. The study sites will strive to keep clinicians informed about the NORD-STAR study to maintain a high rate of enrollment. Recruitment of patients will take place at participating study sites in Denmark, Finland, Iceland, Norway and Sweden in a competitive manner, i.e., all active centers recruit until the goal of 800 patients has been reached. No advertising will be used to recruit patients and participants will not be compensated economically. The patients will receive oral and written information about the study. Patients willing to participate sign a written informed consent and a screening visit is scheduled to ensure that the patients meet the criteria for inclusion. For a complete list of inclusion and exclusion criteria, see Table 2.

**Table 2:** Inclusion and exclusion criteria of the NORD-STAR study

Inclusion criteria
Age ≥18 years
RA according to ACR/EULAR 2010 criteria [55]
< 24 months from arthritis symptom onset
RF positive and/or ACPA positive and/or CRP ≥10 mg/L
DAS28 CRP >3.2 [56]
≥ 2 swollen and ≥2 tender joints (based on a 66/68 joint count) [27]
Female patients are either not of childbearing potential or use birth control (IUD, contraceptives or have a vasectomized partner)
Female patients should have a negative serum or urine pregnancy test upon screening
Judged to be in good general health based on medical history, physical examination, laboratory profile, chest X-ray and electrocardiogram
Willingness to provide written informed consent
Willingness to administer s.c. injections or receive these by trained personnel
Exclusion criteria
Previous DMARD therapy for rheumatic diseases
Current active inflammatory joint disease other than RA
Received prednisone (or equivalent) dose >7.5 mg/day or dose adjustment within the preceding 4 weeks
Received i.a. or parenteral administration of glucocorticoids within the preceding 4 weeks
Undergone joint surgery within the preceding 2 months (at joints assessed in the study)
Diagnosed with chronic arthritis before age of 17 years
History with allergic reactions or significant sensitivity to constituents of study drugs
Treated with any investigatory drugs within the preceding month
Active infection of any kind (except fungal infections of nail beds)
Active infection or infection requiring hospitalization within 4 weeks of screening
Poorly controlled medical condition which, in the opinion of the investigator, would put the subject at risk by participation in the study
History of clinically significant hematologic, liver or renal disease
Neurologic symptoms suggestive of CNS demyelinating disease and/or diagnosis of central demyelinating disease
History of cancer or lymphoproliferative disease (successfully treated cutaneous squamous cell or basal cell carcinoma, localized carcinoma in situ of the cervix and curatively treated malignancy >5 years prior to screening are allowed)
History of listeriosis/histoplasmosis/untreated tuberculosis or persistent chronic infections, or recent active infections requiring hospitalization or treatment with i.v. anti-infectives within 30 days or orally administered anti-infectives within 14 days prior to baseline visit
Latent tuberculosis
Severely immunocompromised
Female patients who are breastfeeding, pregnant or considering becoming pregnant during the study or within 150 days after the last dose of study medication
Male patients who are planning to father a child during the time they are included in the study
Clinically significant drug or alcohol usage during the preceding year
Chronic widespread pain syndrome
Considered by the investigator to be an unsuitable candidate for the study
Unwilling to comply with study protocol
Abnormal screening laboratory results (ASAT/ALAT >1.75 times ULN, positive serum hCG, positive test for hepatitis B and/or C, creatinine levels >2 times ULN, hemoglobin <90 g/L, absolute neutrophil count <1.5 × 10^3^/μL, serum total bilirubin ≥1.5 mg/dL)

*Abbreviations*: *RA* rheumatoid arthritis, *ACR* American College of Rheumatology, *EULAR* European League Against Rheumatism, *RF* rheumatoid factor, *ACPA* anti-citrullinated peptide antibody, *CRP* C-reactive protein, *DAS* Disease Activity Score, *IUD* intra-uterine device, *s.c.* subcutaneous, *DMARD* disease-modifying antirheumatic drugs, *i.a.* intra-articular, *i.v.* intravenous, *ASAT* aspartate transaminase, *ALAT* alanine transaminase, *ULN* upper limit of normal, *hCG* human chorionic gonadotropin

### Patient follow-up

In TP1, the patients are scheduled for regular outpatient appointments every second week for the first 8 weeks (visits at weeks 2, 6 and 20 may be replaced by a telephone contact), followed by successively increasing intervals between each visit (Fig. 5). At each visit, joint counts will be performed by a joint-assessor who is blinded to the patient’s treatment alternative and not otherwise involved in the study. Regular clinical assessments, blood samples, urine samples and patient-reported outcomes will be carried out as listed in Fig. 5.

In TP2, the patient is scheduled for regular appointments with the investigator and joint-assessor, as in TP1. At de-escalation of the study medication, the patient will attend the clinic every second week (visits at weeks 2, 6, 14, 18, 20 and 28 from initiating de-escalation may be replaced by a telephone contact) followed by successively increasing intervals between each visit (Figs. 6 and 7). Regular clinical assessments, blood samples, urine samples and patient-reported outcomes will be carried out (Figs. 6 and 7).

Adherence to allocated treatment is monitored by letting the patients record their use of study drugs in patient diaries provided by the study sites (Finland, Iceland, Norway and Sweden) or by asking the patient at each visit if, and how many doses of, study medication have not been taken since last follow-up visit (Denmark).

### Variables and their assessments

#### Demographic data and physical examination

At screening, demographic data (age, gender, duration of symptoms, disease duration, smoking status, alcohol consumption, medical and surgical history, use of concomitant medication) and physical examination (height, weight, body temperature, respiratory frequency, blood pressure, pulse frequency, electrocardiogram) will be carried out (Figs. 5, 6 and 7).

#### Clinical and laboratory assessments

At all visits (Figs. 5, 6 and 7), tender- and swollen-joint counts will be carried out. In total, 68 joints will be assessed for tenderness (temporomandibular, sternoclavicular, acromioclavicular, shoulder, elbow, wrist, 1st to 5th metacarpophalangeal (MCP), 1st interphalangeal (IP), 2^nd^ to 5^th^ proximal interphalangeal (PIP), 2^nd^ to 5^th^ distal interphalangeal (DIP), hip, knee, ankle, tarsus, 1^st^ to 5^th^ metatarsophalangeal (MTP), 1^st^ IP and 2^nd^ to 5^th^ PIP (toes) joints) and 66 joints will be assessed for swelling (all of the above except the hip joints). CDAI and DAS28-CRP (C-reactive protein) will be based on assessment of 28 joints (shoulder, elbow, wrist, 1^st^ to 5^th^ MCP, 1^st^ IP, 2^nd^ to 5^th^ PIP and knee joints) [27].

Standard-of-care blood samples (blood count, uric acid, creatinine, total bilirubin, alanine/aspartate transaminase, alkaline phosphatase, sodium, potassium, calcium, albumin, cholesterol, triglycerides, CRP and erythrocyte sedimentation rate (ESR, not assessed in Denmark)) will be obtained. At screening, patients are screened for hepatitis B, C and tuberculosis (purified protein derivate (PPD) or Quantiferon test). Furthermore, samples will be analyzed for rheumatoid factor, ACPA, anti-nuclear antibodies (ANA) at screening and at initiation of TP2 and, if clinically indicated, also throughout the study. If ANA is positive, anti-double-stranded deoxyribonucleic acid (dsDNA) will be analyzed. Urine samples will be analyzed for ketones, pH, protein, red blood cells, white blood cells, nitrate and glucose. For women with childbearing potential, urine or serum human chorionic gonadotropin will be analyzed at each visit. Blood and urine samples will be obtained for biobank storage at several visits (Figs. 5, 6 and 7).

#### Patient-reported outcomes

At each visit (Figs. 5, 6 and 7), the patient’s assessment of pain, fatigue and global impact of RA and also the physician’s global assessment of disease activity will be recorded on Visual Analogue Scales (VAS 0–100). Duration of morning stiffness will be recorded. Physical function will be assessed using a Health Assessment Questionnaire (HAQ) [28]. Work-related issues will be assessed using the Work Productivity and Activity Impairment (WPAI) questionnaire [29]. Level of fatigue is assessed using the Functional Assessment of Chronic Illness Therapy (FACIT) fatigue scale [30]. Health-related quality of life is assessed using the EuroQol 5 dimensions (EQ5D) [31] and the Short Form 36 Health Survey Questionnaire (SF36, only performed at certain visits) [32]. Patient Acceptable Symptom State (PASS) will be used to measure the minimal clinical acceptable state of each patient [33].

#### Conventional radiography

Conventional radiographs of the chest will be obtained at baseline to rule out the presence of tuberculosis or other clinically significant conditions. Conventional radiographs of hands and feet will in TP1 be obtained at screening, weeks 24, 48 and 80 should the patient remain in TP1 at this time point. In TP2, conventional radiographs will be obtained at the initiation (not necessary if the patient has undergone the procedure within 12 weeks), after 24 weeks, and at all exits from the study. The radiographs will be analyzed for bone erosion and joint space narrowing using the Sharp van der Heijde (SvH) score [34]. The radiographs will be assessed with known chronology by two independent readers, blinded to all clinical data.

### Outcomes

#### Primary outcomes

The primary clinical outcome in TP1 is the proportion of patients in remission at week 24 according to the CDAI criteria (i.e., CDAI ≤2.8) [35] which will be compared between the four different treatment groups. The primary clinical outcome in TP2 is the proportion of patients in remission according to the CDAI criteria 24 weeks after tapering of study medication was initiated, which will be compared between the two different tapering groups (i.e., week 24 in TP2-A and week 48 in TP2-B). The primary radiographic outcome is the change of the total SvH score [34] after 48 weeks.

#### Secondary outcomes

Secondary outcomes include the proportion of patients in remission at weeks 24 in TP2 according to the CDAI criteria. Furthermore, the following parameters will be assessed at all time points: CDAI/SDAI (Simplified Disease Activity Index) [35]/2010 American College of Rheumatology (ACR)/EULAR [36] remission, ACR20/ACR50/ACR70/ACR-hybrid, DAS28-ESR (values, DAS28-ESR-based disease categories and EULAR responses), DAS28-CRP (values, DAS28-CRP categories and EULAR responses), SDAI/CDAI values, core set variables (66/68 joint count), FACIT fatigue score and VAS fatigue, WPAI, EQ5D, HAQ, SF36, PASS, morning stiffness, patient VAS for disease activity and pain and physician VAS for disease activity.

Secondary radiographic outcomes include (for all relevant time points) change in SvH score, change in SvH erosion score, change in SvH joint space narrowing score, proportion of patients without radiographic progression and reduction of predicted progression according to the published POPERA model [37].

### Statistics

#### Sample size and power considerations

In order to achieve a power of 85% for detecting any difference between the four treatment arms by an overall chi-square test with a type I error of 0.05, a sample size of 724 is required. In order to achieve a power of 90% for an overall chi-square test of any difference between the four treatment arms with a type I error of 0.05, a sample size of 832 is required. In the NORD-STAR study, a sample size of 800 patients has been selected. The power calculation is based on previously published data.

#### Statistical analysis plan

The NORD-STAR study will be conducted according to the modified intention-to-treat (mITT) principle, and the mITT population will be defined as all patients receiving at least one dose of study medication. Demographic information and baseline characteristics of these patients will be summarized by count and percentages for discrete variables and by summary statistics (mean, standard deviation, etc.) for continuous variables.

The percentage of responders at each study visit will be summarized using nonresponder imputation (missing responses will be counted as nonresponse). Continuous measurements will be summarized using both observed and imputed method by study visit. AEs will be summarized by number and percentage of patients experiencing the AE including the rate of AEs normalized by the study drug exposure. Laboratory measurements and vital sign measurements will be summarized by summary statistics and shift tables as appropriate.

The null hypothesis of no difference in TP1 between responder percentages in arms 1, 2, 3 and 4 will be tested using a two-sided analysis of variance (ANOVA) test at a significance level of alpha = 0.05, followed by testing according to Bonferroni-Dunn. Pairwise comparisons of the difference of proportions in CDAI remission are done sequentially in two steps:

- Step 1: csDMARD therapy (arm 1) versus bDMARD therapy (arms 2, 3 and 4), at 0.05/3, two-sided significance level. If there is at least one significant result, go to step 2. Otherwise stop pairwise testing
- Step 2: compare arms 2, 3 and 4 in a pairwise fashion at 0.05/3, two-sided significance level

As a secondary outcome, the results obtained with conventional therapy in Denmark and Finland (using triple csDMARD therapy + glucocorticoid injections) will be compared against conventional therapy in Norway, Sweden and Iceland (using methotrexate + p.o. glucocorticoids), by ANOVA.

In a secondary analysis the noninferiority hypothesis for csDMARD therapy versus bDMARD therapy will be analyzed with an allowable inferiority margin of 5%, and as exploratory analyses with margins of 10% and 20%.

As a secondary “sensitivity” analysis, the results in TP2 will also be calculated for those patients who during the preceding 24 weeks simultaneously were in CDAI remission and had no swollen joints.

### Data registration and monitoring

All data will be collected on standardized paper or electronic Case Report Forms (CRF) as follows: Denmark, electronic CRF (the DANBIO platform [38]); Finland, paper CRF; Iceland, electronic CRF (the ICEBIO platform); Norway, electronic CRF (Viedoc 4); Sweden, paper CRF. Patients included in the study will be identified by their unique patient numbers. The protocol has been approved by the Swedish Data Protection Authority as the trial is coordinated from Sweden. Study investigators will have access to the datasets generated in the study. Protocol, protocol amendments, investigator’s brochure, informed consent and all study-related documents has been reviewed by an Institutional Review Board and a GCP (Good Clinical Practice)-certified person. All participating sites will be monitored before, during and after the trial by GCP-trained personnel in order to ensure compliance with GCP, the protocol and all other applicable regulations.

### Communication between study sites

The coordinating study center in Sweden can be contacted via e-mail or telephone during working hours. Important note-to-file and protocol modifications are communicated to the participating study sites through e-mail newsletters from the coordinating study center and are made available as amendments at the NORD-STAR Internet website.

### Publications

All planned study outcomes, positive as well as negative and inconclusive, will be published in high-impact, peer-reviewed journals, and the manuscript will be written by authors fulfilling the authorship criteria as recommended by the International Committee of Medical Journal Editors.

### Adverse events

An AE is defined as any untoward medical occurrence in a subject or clinical investigation subject administered a pharmaceutical product and which does not necessarily have a causal relationship with this treatment. All AEs will be registered by the investigator at all visits from the administration of the first dose of study drug to 12 weeks following the administration of the last dose of study drug. The investigator will register the date of onset, description of the event, level of severity (mild, moderate or severe), duration and outcome. Potential relationship with study drugs will be assessed. Serious adverse events (SAEs) include death of the patient, life-threatening events, hospitalization of the patient or prolongation of hospitalization, congenital abnormality, spontaneous or elective abortion, persistent or significant disability/incapacity and important medical events requiring medical or surgical intervention to prevent serious outcomes. All SAEs will be reported to the central study coordinating team within 24 h of the site being made aware of the SAE. The central study coordinating team will judge whether the reported event should be judged as an SAE or a suspected unexpected serious adverse reaction (SUSAR). A SUSAR will be reported to all participating sites within 7 days for potential lethal SUSARs and within 15 days for all other SUSARs. The SAEs will be presented in a yearly report to the national health authorities and ethical committees by each national coordinating investigator, in accordance with the GCP guidelines. All AEs and SAEs will be followed until resolution or stabilization.

## Discussion

The NORD-STAR study is, to our knowledge, the first investigator-initiated randomized trial to compare aggressive csDMARD therapy and three bDMARD treatments head to head in early RA, and to compare different methods of de-escalation.

In TP1 of the trial, the efficacy of four different treatments, or modes of action, in early RA are compared. Despite rapid development of new treatments for RA, the optimal treatment choice in the early stage of disease is not clear. Whereas several bDMARDs, with different modes of action, are all effective in early and later stages of disease, only few trials have compared these drugs head to head. In a randomized noninferiority study of established RA, two different TNF-blocking agents, etanercept and adalimumab, were compared and no significant difference was found [39]. In two randomized controlled trials (RCTs) in RA patients with inadequate response to methotrexate, AMPLE and ADACTA, adalimumab was compared to abatacept and tocilizumab, respectively. Adalimumab and abatacept were equally effective in terms of clinical, functional and radiographic outcomes when added to methotrexate [40]. Superior clinical response was achieved with tocilizumab monotherapy compared to adalimumab monotherapy in patients who were intolerant to, or not candidates for, methotrexate use [41]. The ATTEST trial showed comparable clinical results with infliximab and abatacept in RA patients who had inadequate response to methotrexate. However, fewer infections and SAEs were seen in the abatacept group [42].

Several papers have been published with indirect pairwise comparisons or network meta-analyses, using statistical methods to combine data from different RCTs and making comparisons across treatments. In these analyses, most novel antirheumatic drugs, with the exception of anakinra, showed comparable clinical responses in combination with methotrexate [43, 44]. Indirect comparisons, however, have several limitations, and power and precision is dependent on the effective number of trials, sample size and heterogeneity of study populations. In the NORD-STAR trial, different treatments will be compared head to head, and the possibility that one or more treatments will emerge as particularly suitable for induction-maintenance types of therapies could alter the current paradigms for treating RA.

The NORD-STAR protocol compares aggressive csDMARD therapy with three bDMARD therapies with different modes of action; certolizumab-pegol inhibits TNF, abatacept is a selective T-cell co-stimulation modulator and tocilizumab blocks interleukin 6. There are currently other treatment options available with other modes of actions, including rituximab, a monoclonal antibody that targets CD20-positive B-cells, and tofacitinib, a small molecule drug inhibiting Janus kinase (JAK) given orally. Both rituximab and tofacitinib are also effective in RA [45, 46]. An optimal trial design would include these treatments. However, it was not feasible to include additional treatment arms due to sample size considerations (i.e., the current four-armed design required 800 patients to be included). Furthermore, rituximab is administered twice 14 days apart and then again after 6–12 months, which would have been difficult to fit into the study design. Tofacitinib had not been approved by the EMA (European Medicines Agency) and was, therefore, not marketed in the Scandinavian countries at the time when the study was planned.

The trial involves rheumatology centers in five different countries and patients are treated in routine care, according to protocol. The study has been pragmatically designed to ensure that the treatment algorithms used are easily transferable to daily clinical practice. As an example, there are two alternative treatment regimens in the active csDMARD treatment arm due to different treatment practice in the Nordic countries. Triple therapy with methotrexate, sulphasalazine and hydroxychloroquine in combination with aggressive use of intra-articular glucocorticoid injections (treatment arm 1A) is widely used in Finland and Denmark, whereas in Sweden, Norway and Iceland methotrexate in monotherapy combined with orally administered prednisolone and intra-articular glucocorticoid injections is more commonly used (treatment arm 1B). Both treatment strategies have been shown to be more effective than single-DMARD therapy [6, 47, 48]. The route of administration of tocilizumab and abatacept is another example of the pragmatic approach. When subcutaneous formulations became available, the protocol was modified to include both intravenous (i.v.) and s.c. administration routes. The decision was supported by a noninferiority trial where i.v. and s.c. routes of tocilizumab and abatacept administration were equally effective [49, 50].

Treat-to-target and tight control are two well-established principles in management of patients with early RA [13, 51] that are included in the current study design. Patients who do not achieve CDAI remission in TP1 will exit the study. TP2 implies a dynamic strategy where treatment is modified as long as a response target is not reached. The primary goal in this trial is to compare aggressive csDMARD therapy and bDMARD therapies, thus the protocol does not allow for major treatment changes. However, a tight follow-up regimen is applied, and intra-articular injections can be given in swollen joints at the discretion of the physician throughout the trial (although with upper limits). Additionally, after 6 months, sulphasalazine and hydroxychloroquine can be added to methotrexate in treatment arm 1B, if clinically indicated.

In NORD-STAR, the idea is to treat the disease aggressively in the early phase of disease, and a step-down, rather than a step-up, strategy is applied. In the second part of the study two different tapering strategies are compared. In previous trials, withdrawals of biologic therapy were associated with a subsequent disease flare in 50–90% of patients with established disease [20, 22, 23]. Dose reduction, on the other hand, may be applied in patients in a low disease activity state, as shown with etanercept in the randomized PRESERVE and DOSERA trials [21, 23]. In patients with early RA, good response can be maintained in a larger proportion of patients upon discontinuation of anti-TNF agents [26], supporting the idea of a window of opportunity. The AVERT trial even demonstrated that induction therapy with abatacept and methotrexate in early RA resulted in sustained remission in a small proportion of patients up to 18 months after discontinuation of all RA treatment [24]. However, the PRIZE study showed that remission was lost in a substantial proportion of early RA patients upon withdrawal of etanercept [52]. Thus, the concept of induction-maintenance therapy in early RA is still up for debate.

The degree of suppression of disease activity seems to be a predictor for successful tapering of bDMARD drugs. In the HONOR trial, deep remission (DAS28 < 1.98) was associated with maintenance of response after withdrawal of adalimumab [53]. In NORD-STAR, we recruit DMARD-naïve patients with early disease. Sustained CDAI remission, which is a stringent remission criterion, is required to start treatment tapering. The appropriate time point to start de-escalation in patients who are in stable remission is not known. In part 2 of the NORD-STAR study we address this question.

The NORD-STAR study has an open-label design, where both the investigator and the patient know to which treatment arm the patient belongs. However, in order to allow head-to-head comparison between the different treatments arms, blinding in some fashion is essential in order to avoid biased results. This is solved by having the joint counts performed by blinded assessors, not otherwise involved in the study. The joint count is part of the CDAI score, which is the primary outcome. The primary radiographic outcome will be the difference in the SvH score, which will be evaluated using assessors blinded for all clinical and treatment data. Hence, the risk of bias is reduced.

CDAI was chosen as the primary clinical outcome for several reasons. First of all, according to treat-to-target guidelines, remission is the main treatment goal in RA [51]. Secondly, CDAI is a composite score that does not include ESR and CRP [54], which is important since tocilizumab is known to suppress these inflammatory markers irrespective of the clinical response. Furthermore CDAI ≤2.8 is a stringent remission criterion reducing the risk of residual inflammation.

The ultimate goal in RA management is to prevent loss of function. Both inflammation and structural damage contribute to loss of function. In several studies, bDMARD therapies have been shown as particularly efficient in halting radiographic progression. Thus, a primary radiographic endpoint is included in the study.

The trial is conducted as a unique collaboration of rheumatology centers across the Nordic countries. An International Steering Committee comprises rheumatology experts from all the Nordic countries, each with experience from clinical trials. The committee has regular meetings and is jointly responsible for the study design and major decisions regarding the trial. However, each country is separately responsible for funding, regulatory approvals and conduction of the trial. As a result, patient recruitment will be initiated at different time points in the five participating countries.

Karolinska Institute is sponsor of the trial and a central study team is situated in Stockholm. The central study team is, e.g., responsible for handling of SUSARs, producing annual reports for the medical agencies, and for updating the NORD-STAR Internet site. Other responsibilities have been delegated to the national managements.

Feasible data collection within the frame of daily clinical practice has been important in this trial. Therefore, the use of already established clinical tools has been encouraged for CRF purposes. In Denmark and Iceland, the DANBIO/ICEBIO national registers, which are used routinely at all departments of rheumatology across the two countries, serves as the eCRF. Norway use Viedoc, a web-based solution for clinical trial data collection and management that complies with all relevant regulations in North America, Europe and Japan, including 21 CFR Part 11, CSUCI, ICH GCP, CDISC, HIPAA, PuL and EU Annex 11, whereas in Sweden and Finland paper CRFs have been chosen for data collection.

Several spin-off projects have been approved by the International Steering Committee, including use of ultrasonography, magnetic resonance imaging and biomarker studies. A complete list of current spin-off projects is available at the NORD-STAR Internet site and listed in Additional file 2.

Due to the use of unique personal registration numbers of all inhabitants of the Nordic countries, the study data may be linked to national registers on, e.g., cancer, hospitalization, and socioeconomics with the potential to gain additional knowledge about the long-term impact of the different treatments. Also, health economic calculations can be conducted.

In conclusion, the NORD-STAR trial seeks to identify which treatment strategy should be applied in early RA. The trial aims at identifying the best remission-induction therapy, but also to improve de-escalation strategies. The pragmatic design ensures good external validity of the results. Hopefully, NORD-STAR will contribute to improved outcomes, both for individual patients, and for society at large, by reducing direct and indirect costs of treatments.

### Trial status

Recruitment started in December 2012 and the trial is still recruiting.

## Additional files

Additional file 1: A completed SPIRIT Checklist. (PDF 97 kb)

Additional file 2: A complete list of current spin-off projects. (PDF 173 kb)

## Abbreviations

ABA: Abatacept
ACPA: Anti-citrullinated peptide antibody
ACR: American College of Rheumatology
ACT: Aggressive conventional synthetic disease-modifying antirheumatic drug therapy
AE: Adverse event
ALAT: Alanine transaminase
ANA: Anti-nuclear antibody
ANOVA: Analysis of variance
ASAT: Aspartate transaminase
BL: Baseline
CDAI: Clinical Disease Activity Index
CRF: Case Report Form
CRP: C-reactive protein
DAS: Disease Activity Score
DIP: Distal interphalangeal
DMARD: Disease-modifying antirheumatic drugs (bDMARD/csDMARD: biological/conventional synthetic DMARD)
DR: Dose reduction
EMA: European Medicines Agency
EQ5D: EuroQol 5 dimensions
ESR: Erythrocyte sedimentation rate
ET: Early termination
EULAR: European League Against Rheumatism
FACIT: Functional Assessment of Chronic Illness Therapy
GCP: Good Clinical Practice
HAQ: Health Assessment Questionnaire
hCG: Human chorionic gonadotropin
HCQ: Hydroxychloroquine
i.a.: Intra-articular
i.v.: Intravenous
IP: Interphalangeal
IUD: Intra-uterine device
JAK: Janus kinase
MCP: Metacarpophalangeal
mITT: Modified intention-to-treat
MTP: Metatarsophalangeal
MTX: Methotrexate
PASS: Patient Acceptable Symptom State
PIP: Proximal interphalangeal
PPD: Purified protein derivate
p.o.: Per os
RA: Rheumatoid arthritis
RCT: Randomized controlled trial
RF: Rheumatoid factor
s.c.: Subcutaneous
SAE: Serious adverse event
SDAI: Simplified Disease Activity Index
SF36: Short Form 36
SJC: Swollen joint count
SSZ: Sulphasalazine
SUSAR: Suspected unexpected serious adverse reaction
SvH: Sharp van der Heijde
TCZ: Tocilizumab
TJC: Tender joint count
TNF: Tumor necrosis factor
TP: Treatment part
ULN: Upper limit of normal
VAS: Visual Analogue Scale
WPAI: Work Productivity and Activity Impairment
