# Supplementary material for: Head-to-head comparison of aggressive conventional therapy and three biological treatments and comparison of two de-escalation strategies in patients who respond to treatment: study protocol for a multicenter, randomized, open-label, blinded-assessor, phase 4 study
Source: Trials. 2017 Apr 4;18:161. doi: 10.1186/s13063-017-1891-x (PMC5381054; doi:10.1186/s13063-017-1891-x)
Supplement: Supplementary file 2 — A complete list of current spin-off projects. (PDF 173 kb) [file 13063_2017_1891_MOESM2_ESM.pdf]

**Spin-off projects in the NORD-STAR study**

| Project | Conducting site |
|---|---|
| Bone density | Gothenburg, Sweden |
| B-cells | Gothenburg, Sweden |
| Regulatory T-cells | Gothenburg, Sweden |
| Hand strength and functionality | Gothenburg, Sweden |
| The degree of inflammation in joints | Linköping, Sweden |
| Magnetic resonance imaging (MRI) | Copenhagen, Denmark |
| Coagulation-inflammatory pathways | Solna, Sweden |
| Cotinine and smoking status | Solna, Sweden |
| Musculoskeletal ultrasound | Oslo, Norway |
| Physical function and health related quality of life | Oslo, Norway |
